# Deoxynivalenol as a New Factor in the Persistence of Intestinal Inflammatory Diseases: An Emerging Hypothesis through Possible Modulation of Th17-Mediated Response

**DOI:** 10.1371/journal.pone.0053647

**Published:** 2013-01-10

**Authors:** Patricia M. Cano, Julie Seeboth, François Meurens, Juliette Cognie, Roberta Abrami, Isabelle P. Oswald, Laurence Guzylack-Piriou

**Affiliations:** 1 INRA, UMR1331, Toxalim, Research Centre in Food Toxicology, Toulouse, France; 2 Université de Toulouse, INPT, UMR1331, Toxalim, Toulouse, France; 3 INRA, UMR1282 Infectiologie et Santé Publique, Nouzilly, France; 4 INRA, Physiologie de la Reproduction et des Comportements, Nouzilly, France; 5 Universidade Estadual de Londrina, Lab Patologia Animal, Londrina, Brazil; 6 Vaccine and Infectious Disease Organization, University of Saskatchewan, Saskatoon, Saskatchewan, Canada; Universidade de Sao Paulo, Brazil

## Abstract

**Background/Aims:**

Deoxynivalenol (DON) is a mycotoxin produced by *Fusarium* species which is commonly found in temperate regions worldwide as a natural contaminant of cereals. It is of great concern not only in terms of economic losses but also in terms of animal and public health. The digestive tract is the first and main target of this food contaminant and it represents a major site of immune tolerance. A finely tuned cross-talk between the innate and the adaptive immune systems ensures the homeostatic equilibrium between the mucosal immune system and commensal microorganisms. The aim of this study was to analyze the impact of DON on the intestinal immune response.

**Methodology:**

Non-transformed intestinal porcine epithelial cells IPEC-1 and porcine jejunal explants were used to investigate the effect of DON on the intestinal immune response and the modulation of naive T cells differentiation. Transcriptomic proteomic and flow cytometry analysis were performed.

**Results:**

DON induced a pro-inflammatory response with a significant increase of expression of mRNA encoding for IL-8, IL-1α and IL-1β, TNF-α in all used models. Additionally, DON significantly induced the expression of genes involved in the differentiation of Th17 cells (STAT3, IL–17A, IL-6, IL-1β) at the expenses of the pathway of regulatory T cells (Treg) (FoxP3, RALDH1). DON also induced genes related to the pathogenic Th17 cells subset such as IL–23A, IL-22 and IL-21 and not genes related to the regulatory Th17 cells (rTh17) such as TGF-β and IL-10.

**Conclusion:**

DON triggered multiple immune modulatory effects which could be associated with an increased susceptibility to intestinal inflammatory diseases.

## Introduction

Mycotoxins are fungal secondary metabolites that commonly contaminate human food and animal feed. Given their global and frequent occurrence, their stability through the food processing chain [1] as well as their known toxic effects, mycotoxins have become a major concern in Europe and North America. Their effects on humans and domestic animals range from decreased resistance to infectious diseases and growth impairment, to cancer induction and death [2], [3], [4].

Deoxynivalenol (DON) is a mycotoxin of the trichothecenes family mainly produced by *Fusarium graminearum* and *F. culmorum*. Although it is not the most acutely toxic trichothecene, DON is regarded as an important food safety issue since it is the most prevalent trichothecene in wheat, corn, barley and their by-products in Europe and North America [5], [6]. Toxicity of DON has been largely demonstrated for humans and all animal species tested [7] but swine, which are readily exposed to this toxin through their cereal-rich diet, are the most sensitive [8]. Therefore they make an excellent research model for animal and even human exposure to DON due to higher similarities concerning the digestive and the immune system than other animal models [9], [10]. Acute exposure to high doses of DON results in diarrhea, vomiting, leukocytosis, gastrointestinal hemorrhage and ultimately death whereas chronical exposure to low doses of this toxin induces anorexia, reduced weight gain and altered nutritional efficiency [7].

This toxin specifically targets dividing cells such as intestinal epithelial cells and immune cells [11]. It can alter the expression of transcription factors by readily binding to the ribosomes and rapidly activating mitogen-activated protein kinases (MAPKs), thus possibly affecting cytokines production and membrane receptors [12].

The intestinal tract is the first physical barrier against food contaminants, chemicals and intestinal pathogens and as such, it plays an important role in the regulation of the immune response to these intrusions. Previous studies have demonstrated that intestinal absorbance of DON takes mainly place in the jejunum both directly from the intestinal lumen to the apical side of the intestinal epithelium and also indirectly through already absorbed toxin reaching the intestinal epithelial cells (IECs) from the blood stream to the basolateral side [13]. Low doses of this toxin can induce morphological and histological impairments of IECs such as atrophy and fusion of the villi [14], [15]. It can also decrease the number of goblet cells [14] and alter the intestinal permeability by repressing the expression of tight junction proteins such as claudins 3-4, ZO-1 and occludin [14], [16], [17], [18]. Basolateral exposure to DON recently showed to have a more severe impact on the intestinal barrier integrity and DNA fragmentation than apical exposure [19].

As a defense mechanism, the intestinal epithelial cells are able to produce several pro-inflammatory cytokines such as the tumor necrosis factor-alpha (TNF-α), interleukin (IL)-6 and different chemokines such as IL-8 and the CC-chemokine ligand (CCL)-20 that are crucial for the recruitment and activation of the underlying immune cells of the *lamina propria* [20]. IECs also have a particularly close interaction with dendritic cells (DCs) that play an essential role in the initiation of the adaptive immune response mediated for the most part by CD4^+^ T helper (Th) cells. Depending on the local intestinal environment, different populations of DCs shape the immune response by activating naive T cells and inducing their differentiation into specific effector T cell populations [21]. Against intracellular infections, Th1 cells are induced to secrete interferon-gamma (IFN-γ) and IL-12 and regulate cellular immunity. DCs are also strongly implicated in the maintenance of tolerance by inducing T regulatory cells (Tregs) that secrete IL-10 and the tumor growth factor beta (TGF-β). Another subset of T helper lymphocytes, the Th17, was recently described as an important mediator of mucosal immunity, defense against extracellular pathogens and autoimmunity [22]. Interestingly, Th17 cells have been related to both pathogenic and regulatory responses, which are generated by two distinct populations of Th17 with a common signature cytokine, IL-17A [23], [24]. The dichotomy of Th17 cells results from different stimuli: IL-6, IL-1β and IL-23 promote the expression of pro-inflammatory cytokines by the so-called pathogenic Th17 whereas IL-6 and TGF-β restrain the pathogenic potential of these cells by inducing the production of IL-10 by regulatory Th17 (rTh17) [23], [24]. The same plasticity allows induced Tregs to reverse their phenotype and acquire the functions of pathogenic Th17 when cultured with IL-6 [25]. Plasticity of T lymphocytes ensures the adequacy of the immune response to an offensive situation and is critical to maintain the balance between protection and homeostasis. All of this relies on a well-established communication between IECs, DCs and the different T helper cell subsets. The damages caused to the intestinal epithelium by exposure to DON could disrupt these interactions and thus lead to severe disturbances of the intestinal immune system. Taken together, all of this could lead to persistent tissue inflammation and eventually, to chronic intestinal inflammatory diseases such as intestinal bowel disease (IBD) characterized by a dysfunctional intestinal barrier and autoimmune responses. DON could play a role in the induction and/or persistence of such inflammatory diseases [26]. However it is noteworthy that few publications have paid attention to the intestinal effects of DON, and even less to the effect of DON on the above mentioned balance between T cells populations.

The aim of this study was to analyze the influence of a low but relevant concentration of DON on the cytokines levels produced by the different subsets of intestinal T lymphocytes using three methodological approaches: *in vitro*, a non transformed porcine intestinal epithelial cell line (IPEC-1) [16], [17] and e*x vivo* porcine intestinal explants [27].

## Materials and Methods

### Chemicals

Purified DON stock (Sigma Aldrich, Ayshire, UK) was dissolved in dimethylsulfoxide (DMSO) and stored at −20°C before dilution in complete cell culture media. Control samples were treated with equivalent concentrations of DMSO, which were non cytotoxic.

### Treatment of the Intestinal Epithelial Cell Line IPEC-1

The Intestinal Porcine Epithelial cell line (IPEC-1) was derived from the small intestine of a newborn unsuckled piglet [28]. IPEC-1 cells are capable of differentiating into mature enterocytes to form a uniform and polarized epithelial layer that suitably mimics the intestinal epithelial barrier and its apico-basolateral discrimination. They have been largely previously used to study bacterial infections, intestinal epithelial integrity, and trans-epithelial transport [18], [29], [30].

IPEC-1 cells were grown and differentiated as previously described [31]. Briefly, cells were seeded into 4.2 cm^2^ polyethylene terephtalate membrane inserts with 0.4 µm pore size (Beckon Dickinson, Pont de Claix, France) at 2 x 10^5^ cells per well in 2 ml of growth media. Treatments were applied to the apical compartments of the inserts.

IPEC-1 cells were incubated for 1.5 h, 4 h, 8 h, 12 h or 24 h in the presence of 10 µM of DON or DMSO at 39°C in a humidified atmosphere of 5% CO_2_. After treatment, supernatants were collected to evaluate cytokine production and cells were harvested for transcriptional analysis.

### Treatment of Explants Cultures

All animal experiments were carried out in accordance with European Guidelines for the Care and Use of Animals for Research Purposes. Jejunal tissue was obtained from six piglets which were 5 week-old, 7–days after weaning. Animals were fed ad libitum prior to the experiment. A 5 cm middle jejunum segment was collected in pre-warmed Williams media (Sigma) supplemented with 200 U/mL penicillin and 200 µg/mL streptomycin (Eurobio, Courtaboeuf, France). It was washed twice and opened longitudinally. Then, the external *tunica muscularis* was removed and explants were made with punch trocards (centravet, Lapalisse, France) and were placed in Williams culture media supplemented with 1% of penicillin/streptomycin, 0.5% of gentamycine (Eurobio), 4.5 g/L of glucose (Sigma), 10% FBS (Eurobio) and 30 mM of amino acid (Ala/Glu) (Eurobio). Pig jejunal explants were incubated with 10 µM of DON or DMSO for 4 h, 8 h or 12 h at 39°C in a humidified atmosphere of 5% CO_2_. After treatment, tissues were collected for transcriptional analysis.

### IL-8, IL-1 Alpha and IL-17 Cytokine Assays

Concentrations of IL8 and IL-1 alpha were measured by enzyme linked immuno-absorbent assays (ELISA) using specific kits for porcine IL8 and IL-1 alpha (R&D Systems, Minneapolis, MN, USA). Plates were washed with PBS/Tween 20 and then blocked with PBS containing 1% BSA (w/v) for 1 h at room temperature (RT). Supernatant samples were added to the ELISA plate in duplicate and incubated for 2 h at RT. After washing, wells were incubated with biotinylated specific detection antibodies for porcine IL8 and porcine IL1-alpha (R&D Systems) for 2 h at RT. Then, streptavidin-HRP-conjugated antibody (Thermo Fisher Scientific, Courtaboeuf, France) was added for 30 min at 37°C. Positive reactions were revealed by 3,3′,5,5′-tetramethylbenzidine (TMB) (Thermo Fisher Scientific) and reactions were stopped with H_2_SO_4_ 2 N. The optical density (OD) was read at 450 nm.

Cytokine IL-17 was measured using the swine IL-17A VetSet™ ELISA Development kits (Kingfisher biotech, St.Paul, MN, USA).

### RNA Extraction and Quantitative Real-Time Polymerase Chain Reaction (qRT-PCR) Analysis

Quantitative real-time PCR (qPCR) was performed to determine the relative mRNA expression levels of chemokines, interleukins, chemokines receptors, enzymes, and several transcription factors involved in the modulation of the intestinal immune response. Total RNA from IPEC-1 cultures and jejunal explants was extracted with Trizol Reagent (Extract all, Eurobio). RNA concentration, integrity and quality were determined spectrophotometrically using Nanodrop ND1000 (Labtech International, Paris, France). RIN of these mRNA fluctuated between 6.80 and 4.70 for 4 hours and 12 hours of culture times, respectively. Then, reverse transcription and real-time qPCR steps were performed as previously described [32]. Non-reverse transcripted RNA was used as non-template control for verification of genomic DNA amplification signal. Specificity of qPCR products was assessed at the end of the reactions by analyzing dissociation curves. Primers were purchased from Invitrogen (Invitrogen, Life Technologies Corporation, Paisley, UK). Specific sequences were specified in table? 1. Amplification efficiency and initial fluorescence were determined by the ΔΔCt method, then obtained values were normalized using two reference genes, ribosomal protein L32 (RPL32) and cyclophilline A [33]. Stability of these genes was demonstrated previously in intestinal tissues [34]. mRNA expression levels were expressed relative to the mean of the control group at 4 h of exposure to DMSO alone.

**Table 1:** List of genes, primers sequences (F: Forward; R: Reverse) and accession numbers and references.

Gene Symbol	Gene name	Primer sequence	References
IL1-α	Interleukin 1 - alpha	F: TCAGCCGCCCATCCAA	NC_010445.3
		R: AGCCCCGGTGCCATGT	
IL1-β	Interleukin 1 - beta	F: GAGCTGAAGGCTCTCCACCTC	NM_001005149
		R: ATCGCTGTCATCTCCTTGCAC	
TNF-α	Tumor necrosis factor - alpha	F: ACTGCACTTCGAGGTTATCGG	NM_214022
		R: GGCGACGGGCTTATCTGA	[32]
IL6	Interleukin 6	F: GGCAAAAGGGAAAGAATCCAG	NM_214399
		R: CGTTCTGTGACTGCAGCTTATCC	[68]
IL8	Interleukin 8	F: GCTCTCTGTGAGGCTGCAGTTC	NM_213867
		R: AAGGTGTGGAATGCGTATTTATGC	[14]
IL10	Interleukin 10	F: GGCCCAGTGAAGAGTTTCTTTC	NM_214041
		R: CAACAAGTCGCCCATCTGGT	[14]
IL12-p40	Interleukin 12 p40	F: GGTTTCAGACCCGACGAACTCT	NM_214013
		R: CATATGGCCACAATGGGAGATG	
IL17α	Interleukin 17 - alpha	F: CCAGACGGCCCTCAGATTAC	AB102693
		R: CACTTGGCCTCCCAGATCAC	[69]
IL21	Interleukin 21	F : GGCACAGTGCCCCATAAATC	MN_214415
		R: GCAGCAATTCAGGGTCCAAG	[70]
IL22	Interleukin 22	F: AAGCAGGTCCTGAACTTCAC	AY937228
		R: CACCCTTAATACGGCATTGG	[69]
IL23A	Interleukin 23 - alpha	F: GAGAAGAGGGAGATGATGAGACTACA	[66]
		R: GGTGGATCCTTTGCAAGCA	
TGF-β	Transforming growth factor - beta	F: GAAGCGCATCGAGGCCATTC	X54859
		R: GGCTCCGGTTCGACACTTTC	[69]
CCL20/MIP3 alpha	Chemokine (CCL20)	F: GCTCCTGGCTGCTTTGATGTC	NM_001024589
		R: CATTGGCGAGCTGCTGTGTG	[66]
IFN-γ	Interferon - gamma	F: TGGTAGCTCTGGGAAACTGAATG	NM_213948
		R: GGCTTTGCGCTGGATCTG	[66]
ROR-γ like	Nuclear receptor ROR-gamma-like	F: CCTGGCCCTGGGCATGT	NC_010446.3
		R: TGTTCTAGCAGCGTCCGAAGT	
FoxP3	Forkhead box P3	F: GGTGCAGTCTCTCTGGAACAA	AY669812
		R: GGTGCCAGTGGCTACAATAC	[66]
CX3CL1/Fractalkine	Chemokine (CX3CL1)	F: GCAGCTCCTAGTCCATTAC	EST CK464144
		R: CACCATTCTGACCCAAAG	[66]
XCR1	Chemokine (XCR1)	F: TCTTCTGCAAGCTTCTCAACATC	[66]
		R: GGCTGACCACGGACAGGTA	
CCR6	Chemokine (CCR6)	F: CCTGCACTGCTGCCTCAA	[71]
		R: TTCAGAAAGTAGCTCCGGAA	
STAT3	Signal transducer and activator of transcription 3	F: TGCAGCAGAAAGTGAGCTAC	MN_001044580
		R: CCGGTCTTGATGACTAATGG	[22]
RALDH1	Retinaldehyde deshydrogenase 1	F: TGGAGTGTGTGGCCAGATCA	N–010460–3
		R: GCAGGCCCTATCTTCCAAATG	[72]
T-bet	T-cell beta chain Th17-Th22 like	F: TTTGTGGCCTTTTGCATCCT	Present study
		R: CCTGTGTTTGTGATCTTGTTCCTT	
Cyclo A	Cyclophilin A	F: CCCACCGTCTTCTTCGACAT	MN_214353
		R: TCTGCTGTCTTTGGAACTTTGTCT	[22]
RPL32	Ribosomal Protein L32	F: AGTTCATCCGGCACCAGTCA	MN_001001636
		R: GAACCTTCTCCGCACCCTGT	[16]

### Analysis of T Regulatory Cells Phenotype in Lamina Propria Cultured With DON

Leucocytes were isolated from jejunal lamina propria harvested from two pigs by collagenase digestion as described previously [35]. Briefly, pieces of small intestine were incubated for 2 times 45 min in several changes of calcium and magnesium-free Hank's balanced salt solution (HBSS), containing 1 mM of ethylenediamine tetra-acetic acid (EDTA) at 37°C on a shaking platform. This procedure largely removed the epithelial layer. The remaining tissue was then digested in RPMI-1640 containing 0.36 g/ml of collagenase (Collagenase Grade V, Sigma), 10% of FBS, 20 mM of Hepes (Eurobio, Paris, France) and 2000 U/ml of Dnase1 (Roche diagnostics, Mannheim, Germany) at 37°C for 1 h on a shaking platform. Cells were suspended in a 40% Percoll in RPMI solution and underlaid with a 80% Percoll solution (Pharmacia, Uppsala, sweden). Low-density cells were recovered from the 40%/80% gradient interface. Cell suspensions isolated from lamina propria were cultured with different concentration of DON: 0.1 to 10 mM for 48 h at 39°C.

Following 2 days of culture, lymphocytes were evaluated for their expression of CD4, CD25, and FoxP3 using flow cytometry. Briefly, cells were first stained with mouse anti-porcine CD4 (clone MIL17, ACRIS, Herford, Germany) and mouse anti-porcine CD25 (AbD Serotec, Colmar, France). As secondary antibodies isotype-specific fluorochrome- or biotin-labelled goat anti-mouse antibodies (αIgG1-Alexa 488, αIgG2b-biotin (Invitrogen) were used. Biotin-labelled secondary antibodies were further marked with APC-Cy7 streptavidin conjugate (Invitrogen). For intracellular staining, cells were permeabilized with a FoxP3 permeabilization/fixation buffer kit followed by staining with anti-mouse/rat FoxP3-PE that reacts with porcine FoxP3 (eBioscience Inc., San Diego, CA). Flow cytometric analysis was conducted on the lymphocytes using a MACSQuant analyser (Miltenyi biotech, Paris, France) and Venturi solfware. Dendritic cells were excluded based on forward and side scatter.

### Statistics

After checking of normal distribution of data, we performed Fisher test and Student’s t-test to compare values between multiple groups. The statistical analysis of the data was carried out with Statview software (Statistical Analysis System; SAS for Windows 98; SAS Institute Inc., Cary, NC, USA). Differences were considered to be statistically significant when the p-value was lower than 0.05.

## Results

DON potentiated the expression of immune genes and increased protein concentration in differentiated IPEC-1 cells in a time-dependent manner.

We initially aimed to investigate the effects of DON on gene expression and protein production involved in the inflammatory immune response. Characterization of DON immune modulatory effects was first assessed by mRNA expression analysis in intestinal epithelial cells (IPEC-1) (Fig.? 1). In untreated cells, no statistical difference in mRNA expression was measured between the different times of exposure, reflecting the absence of spontaneous inflammation caused by extended culture time. Exposure to DON significantly increased the expression of pro-inflammatory and regulatory cytokines, chemokines and of the enzyme RALDH (Fig.? 1A). Early up-regulation in response to DON treatment was observed for IL-8, IL-1α and CCL20. Expression of these genes reached a maximum level after 4 h of DON exposure (100, 14 and 18 fold increase, respectively). mRNA expression levels of these three cytokines decreased in the course of time but remained significantly up-regulated until 24 h of DON exposure. These responses were correlated with IL-8 and IL-1α protein levels which were significantly up-regulated after 8 h of DON exposure (Fig.? 1B). In addition to these early expressed genes, TGF-β, CX3CL1 and RALDH1 were also significantly increased but only after 8 h of exposure to DON (Fig.? 1A).

**Figure 1:**
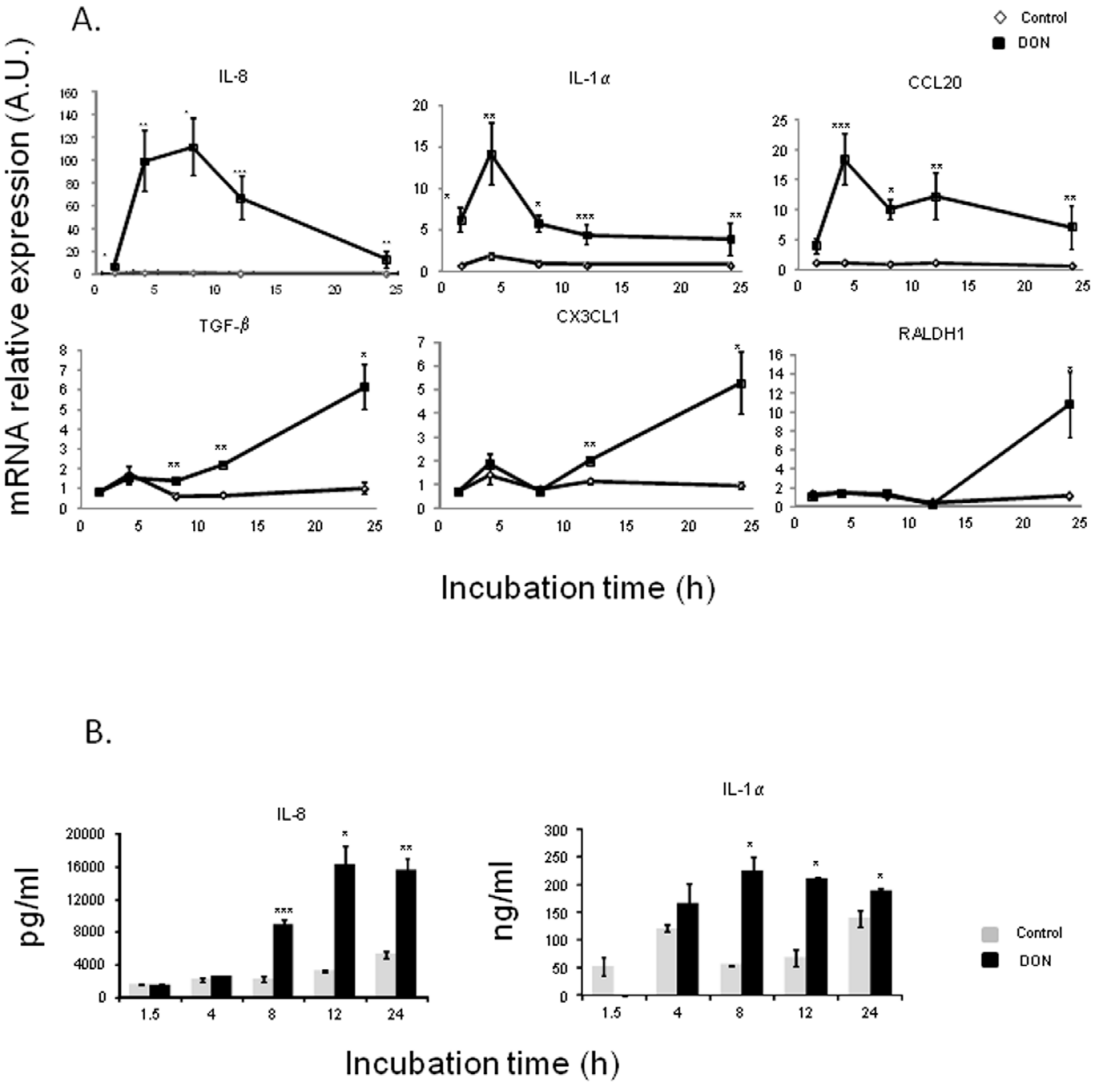
Deoxynivalenol induced cytokines and chemokines mRNA relative expression (A) and protein concentration (B) in IPEC-1 cells .

Differentiated IPEC-1 cells were cultured in presence (black squares/bars) or absence (white diamonds/gray bars) of 10 µM of DON for 1.5 h, 4 h, 8 h, 12 h or 24 h. Gene expression was analyzed by RT-qPCR and protein levels by ELISA. Gene expressions were normalized by the mean of two reference genes (Cyclophilin A and RPL32). Data are presented as means +/− SEM of values obtained with three independent experiments. mRNA values are expressed relative to the control group at 4 h. Asterisks denote significant differences between groups: * P<0.05; **P<0.01; *** P<0.001.

### DON increased relative mRNA Expression Levels of Both pro-Inflammatory Cytokines and DC-Recruiting Chemokines in an *ex vivo* Model of Porcine jejunal Explants

In order to get an insight on the impact of DON on the cross-talk between epithelial cells and immune cells and its impact on the emergence of the mucosal immune response, porcine jejunal explants were exposed to 10 µM of DON. After 4 h, DON exposure induced an up-regulation of mRNA expression of pro-inflammatory cytokines (IL-8, IL-1α and TNF-α) as well as DC-recruiting chemokines (CCL20, CCR6, CX3CL1 and XCR1) (Fig.? 2). Concerning pro-inflammatory cytokines, two different kinetics appeared again. The increase of both IL-8 and IL-1α was progressive in a time dependent manner and these cytokines reached their highest expression levels after 12 h (12 and 13 fold induction, respectively) (Fig.? 2A). On the contrary, the significant up-regulation of TNF-α kept more or less steady along the different time points. An additional experiment involving jejunal loops of a 5 week-old pig *in vivo* to 10 µM of DON, suggested a decrease in the expression of these pro-inflammatory cytokines after 24 h of exposure (Fig. S1).

**Figure 2:**
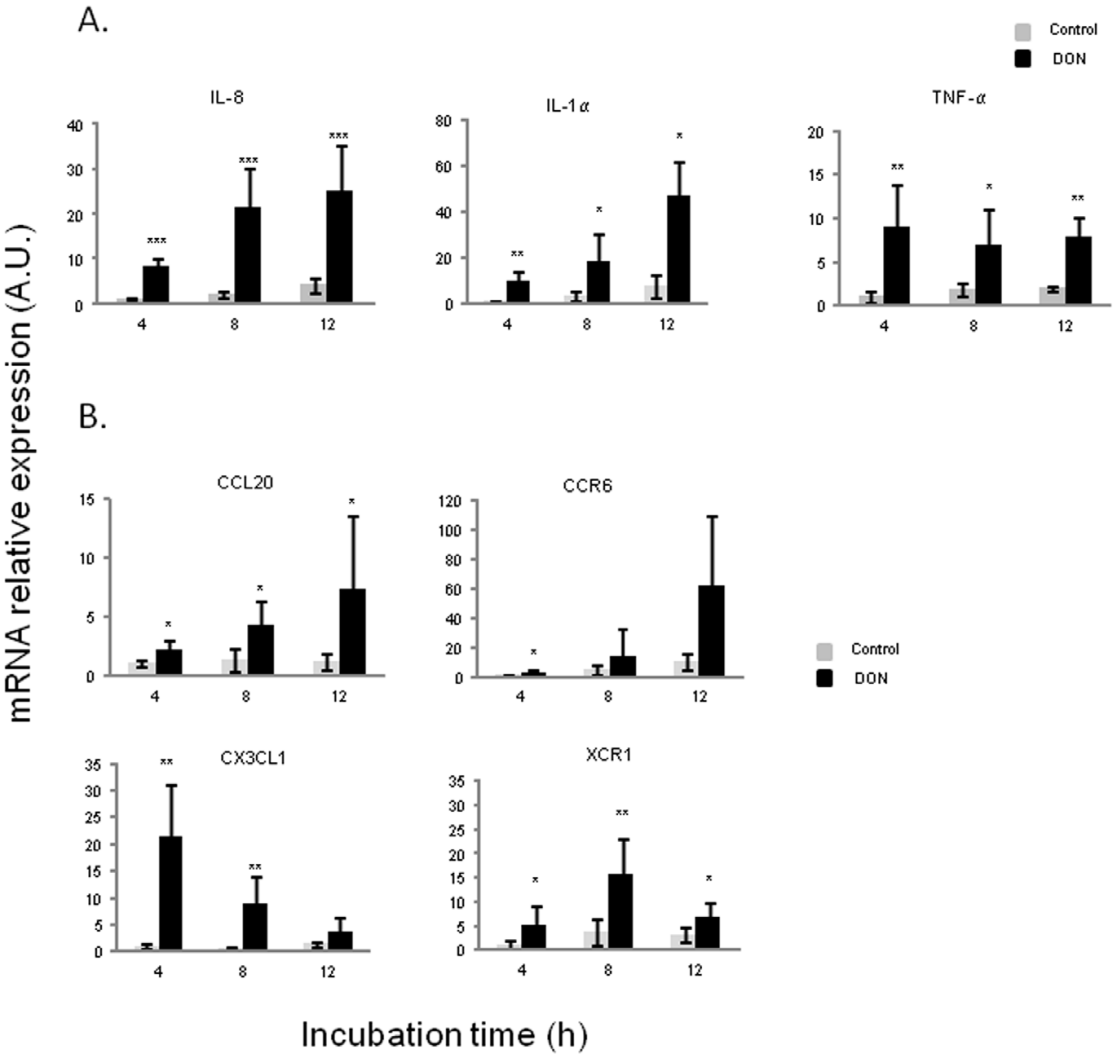
Deoxynivalenol up-regulated pro-inflammatory cytokines (A) and DCs-recruiting chemokines mRNA relative expression (B) in jejunal explants.

The expression of DC-recruiting chemokines was also measured in our explants model to analyze the effect of DON on CCL20/CCR6-mediated signals which can induce chemotaxis of CCR6-expressing DCs and macrophages to sites of tissue damage, driving preferentially the differentiation of naive T-cells towards Th1 or Th17 (Fig.? 2B). The exposure of jejunal explants to DON significantly increased the expression of CCL20 and CCR6 with a peak after 12 h of exposure (7 and 12 fold induction, respectively). Secondly, we analyzed the expression of CX3CL1 which is known to inhibit Th1 and Th17 differentiation. Opposite to CCL20 and CCR6, CX3CL1 exhibited a maximum peak at 4 h (22 fold induction) and decreased progressively after 8 h and 12 h of DON exposure. XCR1, which is also implicated in DC recruitment, was significantly induced by DON at all exposure times but its maximum level was reached at 8 h of DON exposure (7 fold increase). The expression of both CX3CL1 and XCR1 decreased after their maximal induction peak (after 4 h and 8 h respectively) and this tendency was maintained in the loops experiment after 24 h of exposure (Fig. S1).

Explants were exposed or not with 10 µM of DON for 4 h, 8 h or 12 h. Gene expressions were evaluated by RT-qPCR and normalized with two reference genes (Cyclophilin A and RPL32). Data are presented as means +/− SEM of values obtained with intestinal explants from six different piglets and expressed relative to the control group at 4 h. Asterisks denote significant differences between untreated (gray bars) and DON-treated explants (black bars): * P<0.05; **P<0.01; *** P<0.001.

### DON Triggered Th17 Signature genes in Porcine jejunal Explants

In order to examine the effects of DON on naive Th cells differentiation, we analyzed mRNA expression levels of transcription factors and cytokine genes related to Th1 cells (Fig.? 3), Th17 cells (Fig.? 4) and Treg cells (Fig.? 5). DON treatment did not affect expression of T-bet transcription factor nor IFN-γ mRNA levels (Fig.? 3). Significant increase of IL-12 expression levels was observed at all exposure times (between 6 and 8 fold induction) (Fig.? 3). DON treatment also induced a time-dependent increase of expression of mRNA encoding for IL-17 A, IL-6 and STAT3 (Fig.? 4A). Accordingly the protein expression of IL-17A was significantly increased after 12 h of exposure to DON (Fig.? 4B). IL-1β expression levels were also significantly up-regulated but its levels did not did not vary much in a time dependent manner (16 to 12 fold induction) (Fig.? 4A). Surprisingly, RORγ-like, an important Th17 transcription factor was negatively regulated by DON treatment (Fig.? 4A). A different expression profile was observed in genes related to Treg cells. FoxP3 and RALDH1 were not affected by DON treatment after 4 h and 8 h of culture and they were down regulated after 12 h of toxin exposure (Fig.? 5A). This decrease of Treg cells was also found when analyzing the effect of DON on CD4^+^ CD25^+^ FoxP3^+^ leukocytes extracted from the intestinal lamina propria (Fig.? 5B). 10 µM of DON significantly reduced the CD4^+^ CD25^+^ FoxP3^+^ cell population of 40%. On the contrary, the preliminary *in vivo* results using the loops experiment suggest an increase of expression of Treg cells related genes (Fig. S1).

**Figure 3:**
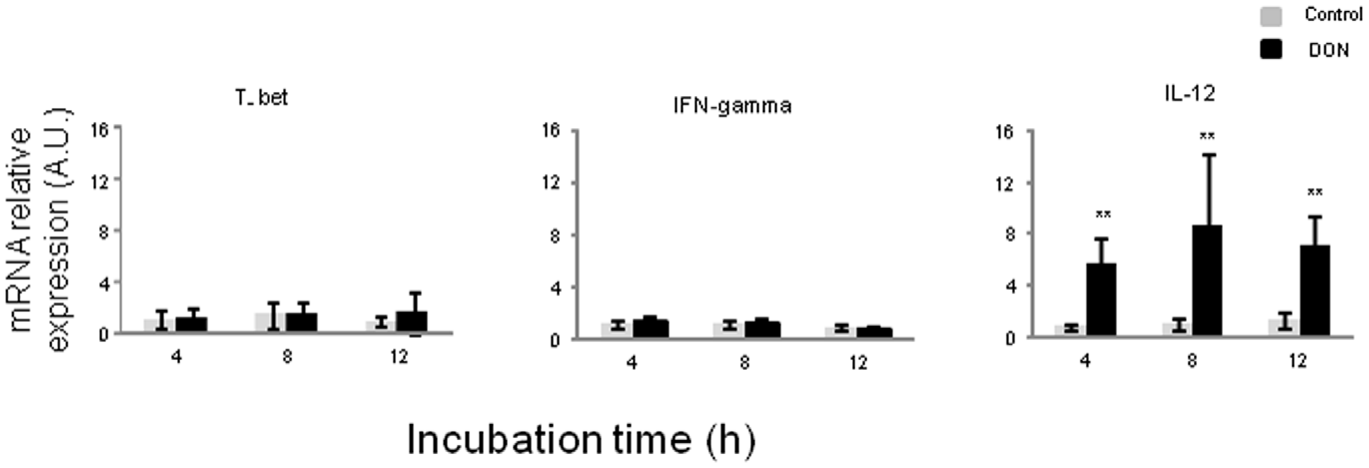
Deoxynivalenol increased IL-12 but not IFN-γ gene expression in jejunal explants.

**Figure 4:**
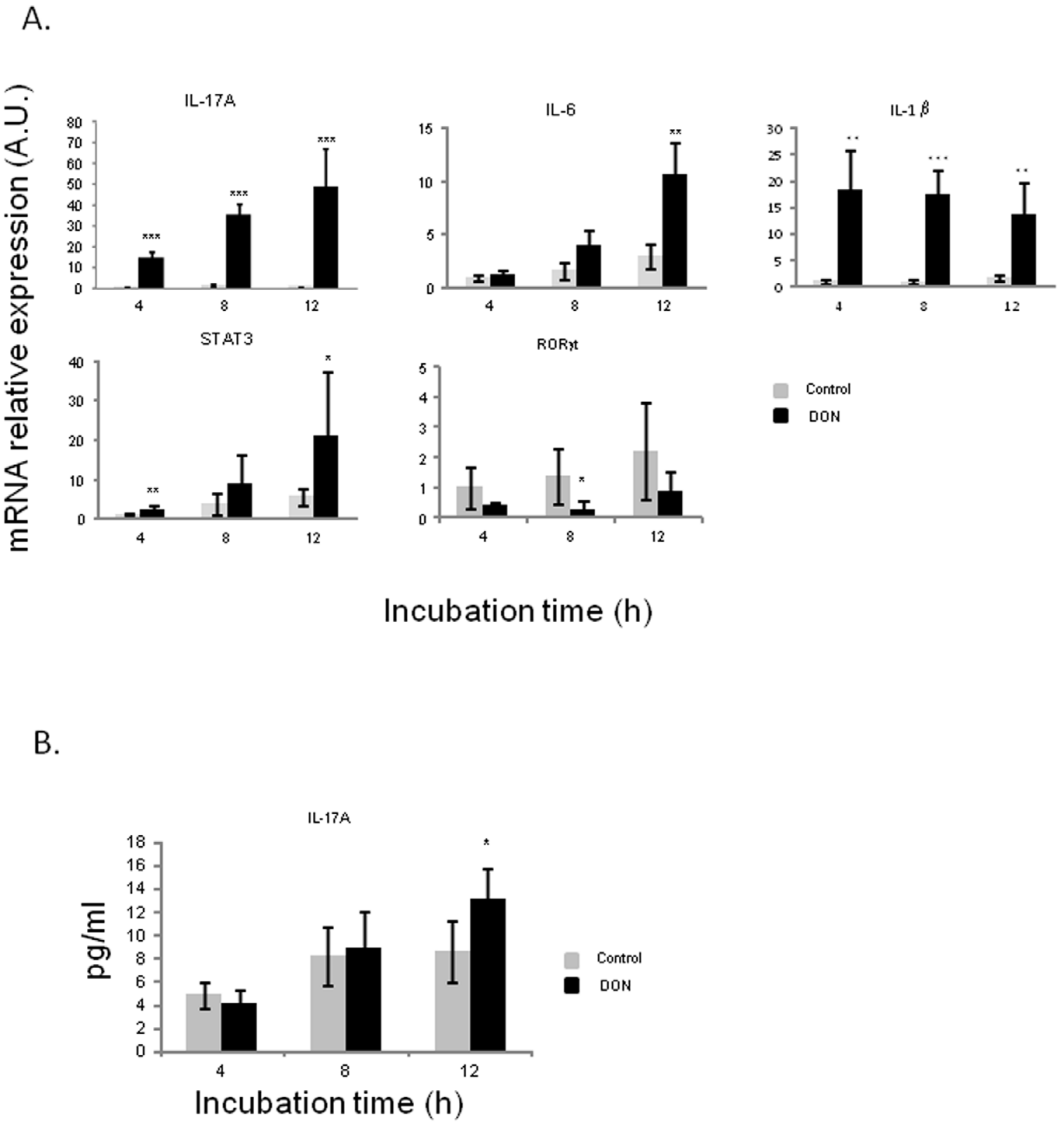
Deoxynivalenol triggered Th17 response in jejunal explants.

**Figure 5:**
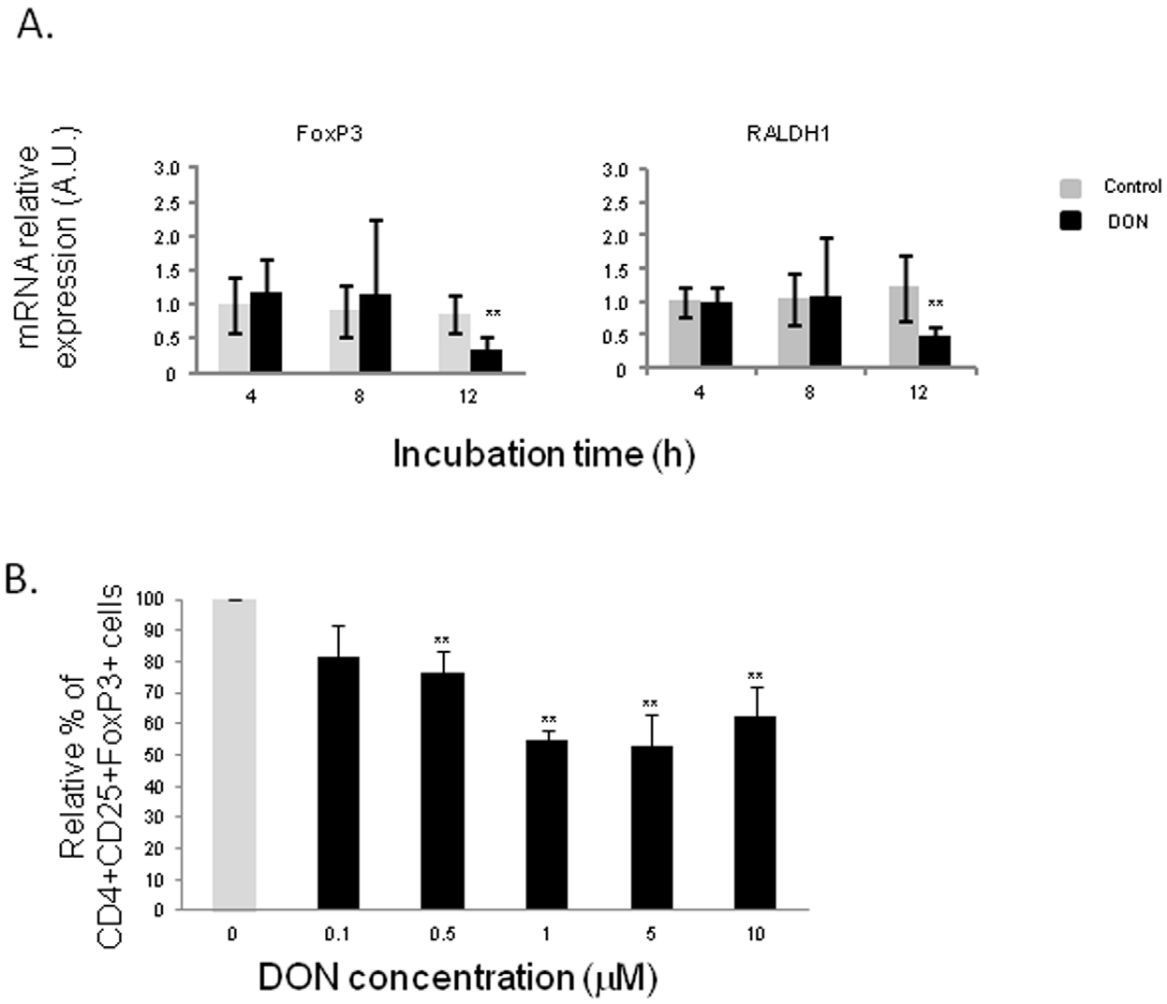
Deoxynivalenol decreased T regulatory response in jejunal explants.

Relative mRNA expression of transcription factors and cytokines related to Th1 cells were assessed by RT-qPCR. Explants were exposed or not with 10 µM of DON for 4 h, 8 h or 12 h. Values were normalized with two reference genes (Cyclophilin A and RPL32). Data are presented as means +/− SEM of values obtained with intestinal explants from six different piglets and expressed relative to the control group at 4 h. Asterisks denote significant differences between untreated (gray bars) and DON-treated explants (black bars): * P<0.05; **P<0.01; *** P<0.001.

Relative mRNA expression of transcription factors and cytokines related to Th17 cells (A) and IL-17 A protein concentration (B) were assessed by RT-qPCR and ELISA. Explants were exposed or not with 10 µM of DON for 4 h, 8 h or 12 h. Values were normalized with two reference genes (Cyclophilin A and RPL32). Data are presented as means +/− SEM of values obtained with intestinal explants from six different piglets and expressed relative to the control group at 4 h. Asterisks denote significant differences between untreated (gray bars) and DON-treated explants (black bars): * P<0.05; **P<0.01; *** P<0.001.

(A) Relative mRNA expression of transcription factors related to T reg cells were assessed by RT-qPCR. Explants were exposed or not with 10 µM of DON for 4 h, 8 h or 12 h. Values were normalized with two reference genes (Cyclophilin A and RPL32). Data are presented as means +/− SEM of values obtained with intestinal explants from six different piglets and expressed relative to the control group at 4 h. (B) The frequency of CD4^+^CD25^+^FoxP3^+^ Treg cells were evaluated by flow cytometry after 2 days of co-culture with different concentration of DON (0.1 to 10 µM). Graphs show the mean ± SEM from duplicates from immune cells of jejunal lamina propria isolated 2 different pigs. Data were calculated as relative percentage to control without toxin. Asterisks denote significant differences between untreated (gray bars) and DON-treated explants (black bars): * P<0.05; **P<0.01; *** P<0.001.

### DON Triggered Pathogenic Th17 Cells Subset in Detriment of Regulatory Th17 (rTh17) Cells in Porcine jejunal Explants

To understand which subset of Th17 cells is preferentially induced by DON, pathogenic Th17-related genes (IL-23 A, IL-22 and IL-21) and rTh17-related genes (TGF-β and IL-10) were analyzed (Fig.? 6). DON significantly increased IL-23A mRNA expression in a time-dependent manner (3, 17 and 31 fold induction at 4, 8 and 12 hours respectively) (Fig.? 6A). mRNA expression levels of both IL-22 and IL-21 were also significantly induced but to a lesser extent (maximum levels of 10 and 3.5 fold induction respectively) (Fig.? 6A). Interestingly, upregulation of these genes did not show after 24 h of DON exposure (Fig. S1). On the contrary, TGF-β mRNA expression was not induced by DON treatment (Fig.? 6B). Interestingly, IL-10 which is produced by both rTh17 and Treg cells showed a significant induction in the presence of DON (6 fold increase) (Fig.? 6B).

**Figure 6:**
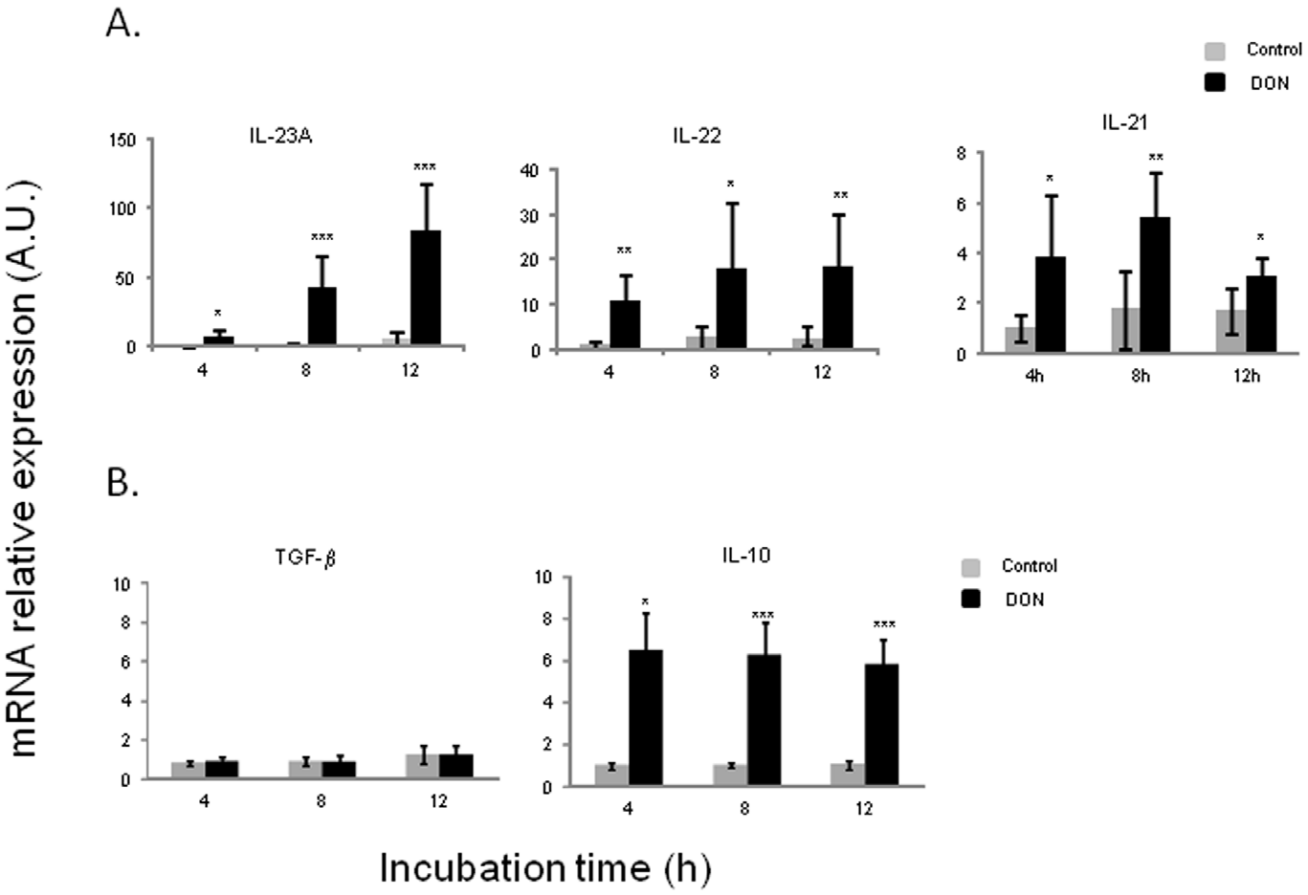
Deoxynivalenol triggered pathogenic Th17 cells subset but not regulatory Th17 (rTh17) cells in porcine jejunal explants.

## Discussion

Mycotoxins have become an issue of major concern given their global and frequent occurrence as well as their known toxic effects. Among all, DON is of particular interest because it is one of the most prevalent mycotoxins in Europe and North America. The intestinal tract represents the first barrier against DON-contaminated food and feed. Many authors have already reported Th17 cells to be critical for protection against microbial infections like bacteria, virus and fungi at mucosal surfaces [36], [37], [38]. The aim of our work was to assess the impact of DON exposure onto the intestinal homeostasis controlled by pro-inflammatory Th17 and the regulatory Treg response. To the best of our knowledge this is the first study showing that DON disrupts the intestinal homeostasis and promotes the Th17 response over the Th1 and T regulatory responses.

Intestinal epithelial cells and jejunal explants were used to investigate the modulation of the intestinal immune response by DON. The choice of using 10 µM of DON was based mainly on the US Federal Grain Inspection Service and the European Union surveys [6], [39], [40] which evaluated the naturally occurring concentration of this mycotoxin to range between 19 and 50 mg/kg in grain cereals. Assuming that DON would be ingested in one meal, diluted in 1 L of gastrointestinal fluid and would be totally bioaccessible, 10 µM correspond approximately to 3 mg/kg [39], [40]. In addition to that, a previous study of our group showed that this concentration does not affect cell viability up to 48 h after treatment in the porcine intestinal cell line, IPEC-1 [17] but it does affect the morphology of the epithelial barrier according to another study [27].

In the present experiment, *in vitro* and *ex vivo* exposure to DON led to an early general state of inflammation depicted by mRNA expression increase of IL-8, IL-1α and TNF- α, which is in agreement with previous results of similar studies [41], [42]. The inflammation generated at the epithelial level could elicit the activation of the population of Th17 cells by triggering further communication with immune cells of the *lamina propria*. The mode of action by which DON induces such inflammatory response has been described by several authors and it was not the purpose of the present study. Indeed, it has already largely been demonstrated that DON activates ERK1/2 and p38 thus triggering MAPK signaling cascades which upregulate COX-2 and PGE-2, major inductors of the inflammatory response [11]. This molecular mechanism most likely occurs in all immune cellular types, including IECs. Therefore we can also soundly hypothesize that DON-induced MAPK activation in IECs induces chemokine secretion which then stimulates intestinal DCs, thus initiating the classical immune response cascade and lymphocyte activation.

In this study, the exposure to DON induced IL-6, IL-23 and IL-1β expression but did not affect the expression of TGF-β and strongly repressed FoxP3 and RALDH1. These data suggest that in our model DON mainly drives the intestinal immune system towards a Th17 response. In agreement with that, DON strongly induced IL-17 A which is the signature cytokine of these T helper cells, but which can also be produced by other immune cells. However to our knowledge, there is no available antibody for of porcine IL-17 A intracellular labeling which would allow to clarify the source of this cytokine. Despite that, the fact that STAT3, a transcription factor expressed by Th17 cells, was also upregulated in the intestinal explants is a strong indication of the increase of this population in the intestinal tissues. Nevertheless, ROR-γt like, which has been described as the other major transcription factor governing Th17 differentiation in mice [23], [43], was not induced but repressed by the presence of DON in explants model. Although this result is surprising, it could be possible that, unlike in the mouse model, in the porcine specie the transcription factor STAT3 plays a more important role than ROR-γt in Th17 differentiation. It would be very interesting to further investigate this point.

The two lineages, Th17 and Tregs share a co-evolutionary origin [44], [45] which could explain why they share similarities at many levels [46], [47], [48], [49]. In this way, CCR6 appears to be the only chemokine receptor essentially implicated in driving Th17 migration *in vivo* but it is also highly expressed by Tregs [50]. CCL20, the ligand of this receptor drives the conversion of the pathogenic Th17 into the regulatory subsets [23], [51]. Our results showed that CCR6 and CCL20 were induced by DON in a time-dependent manner in the intestinal explants. It is therefore tempting to speculate that along the overall pro-inflammatory response both in the intestinal epithelial cells and the intestinal explants, there is a regulatory rTh17 response setting up-rather than the Treg response.

Plasticity of T cells is also reflected in the similarities between rTh17 and Treg cells. Besides their common regulatory action, these two populations share the importance of TGF-β and IL-10 [23], [24], [51]. TGF-β is involved in the differentiation of these two populations. In this study, there was no significant effect of DON on the expression of this cytokine in the intestinal explants. TGF-β was however secreted by the intestinal epithelial cells after 8, 12 and 24 h of exposure to DON. Further studies will be important to understand the exact role of TGF-β in the differentiation of Tregs in swine but also the impact of DON on the intestinal secretion of this cytokine. However, previous studies have also showed that TGF-β presents minor or absent changes after PMA/Ionomycin or CD3 stimulation [52], [53] which might be an indication of the fact that TGF-β might be of less importance in the regulatory response compared to humans and mice. IL-10 is produced by both Tregs and rTh17 to regulate inflammation and has the ability of reversing the pathogenic phenotype of Th17 into rTh17 [24]. In this study, this cytokine was up-regulated in a time independent manner by the presence of DON on explants, which is in contradiction with the absence of increase of FoxP3 and TGF-β. However it is well described that many cells of the innate and adaptive immune system other than Tregs and rTh17 cells express IL-10, such as DCs, macrophages, Th1, Th2 and B cells [54]. Given our results in the intestinal explants on Th1 and Treg, it is most likely for IL-10 to be produced by DCs, macrophages or B cells in that experiment

Taken together, all these results suggest that DON preferentially induces a pro-inflammatory response directed by pathogenic Th17, when CCR6, CCL20 and IL-10 results point to a concomitant protective response most likely lead by rTh17. Supplementary *in vivo* data seems to indicate an increase in the protective response after 24 h of exposure.

Determining whether immune activation or suppression should occur in response to a given pathogen is a critical decision to be taken by the adaptive immune system. Indeed, there is a fine line between inflammation and pathogen elimination and excessive inflammation and tissue damage. Numerous diseases such as rheumatoid arthritis and multiple sclerosis arise from a failure to restore the state of equilibrium [55]. In the intestine, inflammatory bowel diseases (IBD) like ulcerative colitis (UC) and Crohn’s disease (CD) are triggered by excessive inflammation of the colon and/or the small bowel leading to recurrent diarrhea and pain [56]. Until recently only excessive Th1 and Th2 responses were accounted for these diseases. Nevertheless, the discovery of the Th17 subset gave a new insight on the causes of these disorders as IL-17 producing cells were shown to be of major importance in IBD [57]. Besides IL-17, IL-21, IL-22 and IL-23 have also been reported to play an important role in the onset of these diseases [57], [58]. Interestingly, all of these cytokines were up-regulated by the presence of DON in our experiments which could imply that chronic exposure to this toxin could be a triggering or enhancing factor of IBD. This hypothesis is supported by a recent study [26] which draws a strong parallel between the alterations caused by exposure to several mycotoxins including DON and the symptoms observed during IBD. In addition to the induction of the above-mentioned cytokines, intestinal permeability is also related to both DON exposure and IBD. This mycotoxin specifically targets claudin expression [16], [17] which directly leads to an increase of permeability of the intestinal barrier which could result in increased bacterial translocation, one of the main causes of IBD [56]. The same applies for toll-like receptors (TLRs) impairment, associated with DON exposure [59]. Over activation of dendritic cells (DCs) drawn by abnormal TLR activity could lead to inability to detect bacterial components which is another major source of IBD. Notably, TLR activation disruption has been detected after exposure to T-2 toxin, another member of the trichothecene family [60].

As depicted above, bacterial interactions with the immune cells of the *lamina propria* are a central component of IBD, since inflammation might arise from lack of tolerance to antigens present in commensal bacteria [61]. Besides, loss of the transcription factor T-bet, which regulates TNF-α production, influences bacterial homeostasis by favoring colitogenic microbial populations. The resulting increase of TNF-α production by DCs could trigger breakdown of the intestinal epithelium and facilitate bacterial translocation to the *lamina propria*, increasing risks of IBD [62]. It has already been demonstrated that DON alters the intestinal microbiota of pigs [63]. It thus comes as no surprise that in this study, exposure to DON could possibly lead to induction of colitogenic populations and to strong impairment of the epithelial barrier by the lack of effect on T-bet and significant increase of the expression levels of TNF-α. It is noteworthy that colonization of the small intestine by segmented filamentous bacteria (SFB) of the intestinal microbiota is directly associated with the appearance of Th17 cells and that SFB promote the expression of inflammatory genes linked to IBD [64].

In conclusion, exposure to the mycotoxin DON clearly induced an early intestinal inflammatory response resulting from the interplay of different intestinal cell types and leading to the activation of Th17 cells. These results together with previous observations strengthen the idea that chronical exposure to deoxynivalenol could impair intestinal homeostasis and trigger the appearance of IBD. However, the molecular mechanisms by which this toxin specifically activates Th17 remain unclear. Further research should therefore address the link between MAP kinases activation by DON [11], [12] and the herein presented effects on lymphocyte populations. In addition to that, the cellular interplay that takes play among immune cells (IECs – DCs – T cells) upon DON exposure should be better characterized by analyzing for instance cellular migration of DCs, macrophages and T lymphocytes. Finally, more insight should be gained on the role of commensal and pathogenic bacteria on modulating the Th17 response and the impact of DON on such modulation.

## Supporting Information

Figure S1 Fold mRNA expression increase of cytokines and transcription factors after DON exposure in an *in vivo* model of jejunal loops. Following the guidelines provided by the French Council for Animal Care (permit number 2011–07–1) and previous published protocols [65], [66], [67], loops were performed in a 5 week-old pig. Very briefly, 6 consecutive loops (10-–0 cm long) were made by surgical ligation in a 2–4 m long segment of the jejunum which was previously thoroughly washed with a solution of metrodinazole, a common antibiotic. PBS was injected in 3 loops as negative control and 10 µM of DON were injected in 3 other loops. The pig was euthanized by barbiturate overdose 24 h post surgery. Jejunal tissues were then collected and snap-frozen in liquid nitrogen before RNA extraction. Relative mRNA expression levels of immune genes related to pro-inflammatory cytokines (A), DC-recrutment chemokines (B), Th1 (C), Th17 (D) and Treg (E) signature and pathogenic/regulatory Th17 cytokines (F) were assessed by RT-qPCR. Gene expressions were normalized by the mean of two reference genes (Cyclophilin A and RPL32). Data are presented as mean +/− SEM of values obtained with three different loops and expressed relative to the control group. Significant differences between untreated loops (gray bars) and treated loops (black bars) with 10 µM of DON are marked with asterisks (* P<0.05).
(TIF)
