# Supplementary figures and images for: Deoxynivalenol as a New Factor in the Persistence of Intestinal Inflammatory Diseases: An Emerging Hypothesis through Possible Modulation of Th17-Mediated Response
Source: PLoS One. 2013 Jan 10;8(1):e53647. doi: 10.1371/journal.pone.0053647 (PMC3542340; doi:10.1371/journal.pone.0053647)

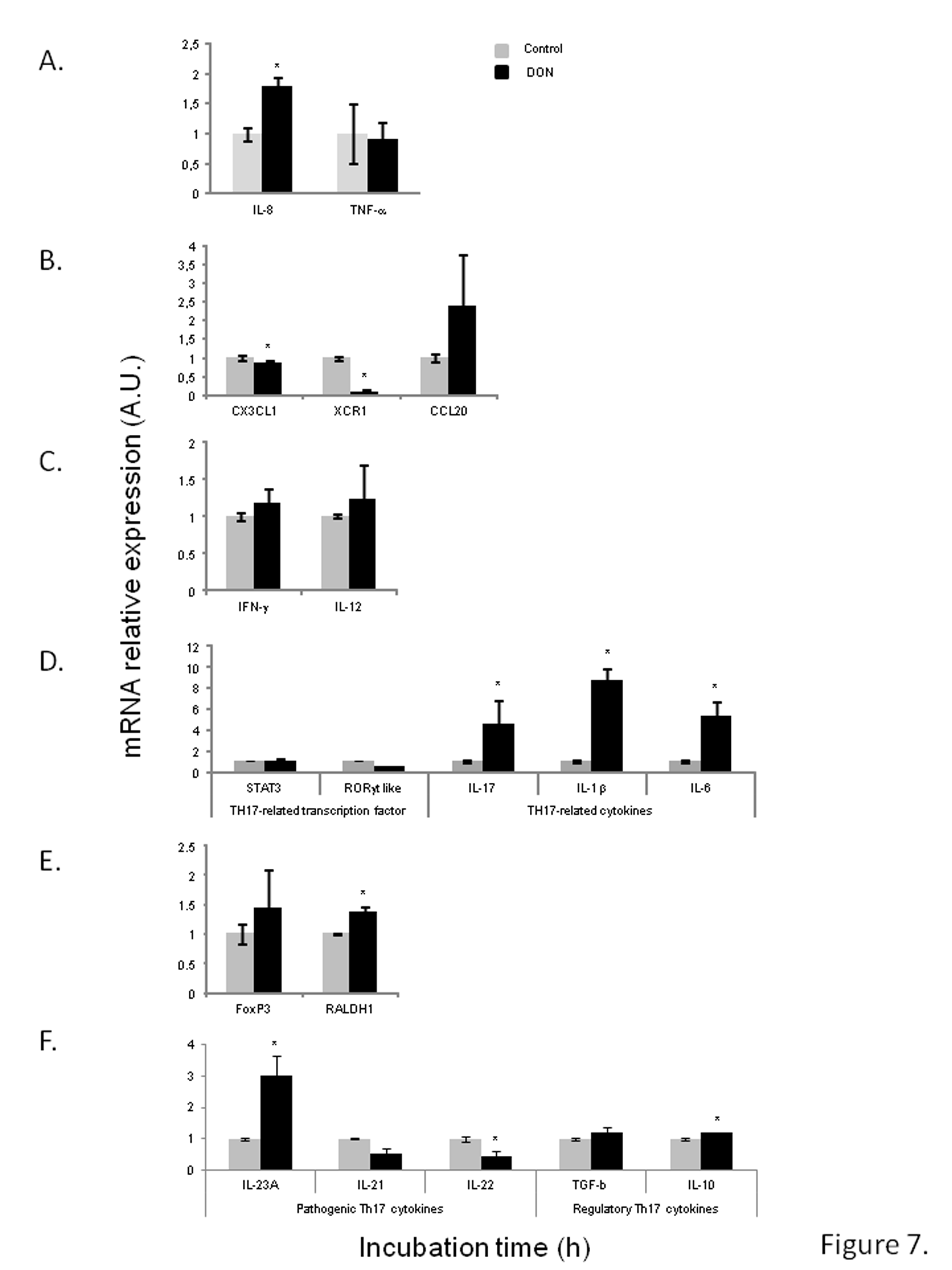

Supplement: Figure S1 — Fold mRNA expression increase of cytokines and transcription factors after DON exposure in an in vivo model of jejunal loops. Following the guidelines provided by the French Council for Animal Care (permit number 2011–07–1) and previous published protocols [65], [66], [67], loops were performed in a 5 week-old pig. Very briefly, 6 consecutive loops (10-–0 cm long) were made by surgical ligation in a 2–4 m long segment of the jejunum which was previously thoroughly washed with a solution of metrodinazole, a common antibiotic. PBS was injected in 3 loops as negative control and 10 µM of DON were injected in 3 other loops. The pig was euthanized by barbiturate overdose 24 h post surgery. Jejunal tissues were then collected and snap-frozen in liquid nitrogen before RNA extraction. Relative mRNA expression levels of immune genes related to pro-inflammatory cytokines (A), DC-recrutment chemokines (B), Th1 (C), Th17 (D) and Treg (E) signature and pathogenic/regulatory Th17 cytokines (F) were assessed by RT-qPCR. Gene expressions were normalized by the mean of two reference genes (Cyclophilin A and RPL32). Data are presented as mean +/− SEM of values obtained with three different loops and expressed relative to the control group. Significant differences between untreated loops (gray bars) and treated loops (black bars) with 10 µM of DON are marked with asterisks (* P<0.05). (TIF) [file pone.0053647.s001.tif]
